# Chronobiological Regulation of Alcohol Intake

**Published:** 2001

**Authors:** Susanne Hiller-Sturmhöfel, Paul Kulkosky

**Affiliations:** Susanne Hiller-Sturmhöfel, Ph.D., is a science editor of Alcohol Research & Health. Paul Kulkosky, Ph.D., is a professor of psychology in the Department of Psychology, University of Southern Colorado, Pueblo, Colorado

**Keywords:** circadian rhythm, biological regulation, light, time of day, AOD (alcohol or other drug) use pattern, pineal gland, melatonin, cholecystokinin, animal model, laboratory rat

## Abstract

Like other physiological functions, food intake and metabolism (including alcohol consumption) in humans and animal models may be regulated by circadian rhythm. For example, many studies of rodents have found that alcohol consumption in these nocturnal animals peaks during their active dark period. This alcohol consumption pattern can be influenced, however, by experimental manipulation. One factor that has been proposed to play a role in regulating circadian alcohol consumption pattern is the hormone melatonin, which is produced by the pineal gland. Research also indicates that the effects of lighting conditions on the alcohol consumption of animal models may be influenced by the differences among the strains of the laboratory animals used, variations in the type and administration schedule of the animals’ alcohol-containing diet, disruptions of the normal circadian rhythm, concurrent use of other drugs, and properties of the light.

The activity patterns and body functions of humans (like those of other animals) are, at least in part, regulated by general environmental influences, such as temperature and lighting conditions. In particular, the daily light-dark cycle (also called the diurnal cycle) has shaped the activity patterns of most animals over millions of years of evolution. Thus, humans generally are active during the daylight and rest during darkness (although modern technology, such as the invention of electrical light, has vastly modified those natural activity patterns). Conversely, rodents are primarily nocturnal animals. These general activity patterns influence numerous other behaviors, including food consumption and metabolism. Accordingly, alcohol consumption patterns in humans and laboratory animals may be affected by the circadian^1^ (i.e., lasting approximately 24 hours) rhythm and the daily light-dark cycle.

This article first describes briefly how researchers assess the alcohol consumption patterns of laboratory animals across the diurnal cycle. It then reviews the influence of daily lighting conditions on the alcohol consumption of rodents and explores the biological mechanisms that may underlie these influences. Finally, the article presents some of the factors that influence the relationship between lighting conditions and alcohol consumption. This discussion briefly describes the implications of these studies for alcohol consumption patterns in humans, particularly in people whose regular circadian rhythm is frequently disrupted (e.g., shift workers or people traveling across different time zones). Many of the topics discussed in this article generated considerable interest primarily in the 1970s and 1980s but, despite their potential relevance to human alcohol consumption patterns, have not yet been thoroughly pursued. As a result, at least part of the literature reviewed in this article is relatively old, although this does not negate its validity.

## ASSESSING DIURNAL DRINKING PATTERNS

To determine the influence of lighting conditions and diurnal drinking patterns of laboratory animals, researchers must regularly monitor the animals’ water (and/or alcohol) consumption. Some studies in this area have focused on the animals’ overall fluid consumption per day under various lighting conditions (e.g., normal light-dark cycles, continuous darkness, or continuous light). For these experiments, the animals have constant access to drinking bottles filled with a specific amount of water and/or an alcohol solution, and their fluid consumption is measured one or more times per day.

When the goal of the experiment is to determine in detail the animals’ consumption patterns throughout the day, however, such an approach is not adequate, particularly if such measurements are to be conducted over several days. Moreover, several bottle changes per day would require waking up the animals periodically, which by itself could influence their drinking behavior and thereby confound the results. Therefore, scientists have developed various types of apparatus that continuously measure fluid consumption and record that information either on a paper printout or, more recently, on a computer. For example, Eriksson (1972) designed a device that measures the fluid level in a laboratory drinking bottle using an electrical probe that is immersed in the bottle and which records specific changes in signal voltage as the fluid level in the bottle decreases. Other investigators have used specially designed cages and drinking bottles that are connected to an electrical circuit and which generate a signal each time the animal licks at the spout of the bottle to receive fluid (e.g., Freund 1970; Dole et al. 1983). Especially with the use of computers to automatically collect, store, and analyze the data, the most current devices can provide investigators with an accurate picture of the times and amounts of fluid consumed by each animal under various environmental conditions. As a result, techniques exist today that allow simultaneous microstructural analysis of the animals’ alcohol, fluid, and food intake (Boyle et al. 1997; Reidelberger et al. 1996). These newer techniques are much more precise and accurate than the earlier procedures previously described and often reveal differences as well as similarities in how and why rats consume alcohol in comparison with humans (Dole et al. 1985; Sinclair 1980).

Some researchers have noticed that the general pattern of food and fluid consumption in rodents resembles a 24-hour sinusoidal curve, with peak levels of consumption around the middle of the dark phase and the lowest (i.e., trough) levels around the middle of the light phase. Based on this observation, investigators can generate a curve that reasonably reflects the animals’ food and fluid (including alcohol) intake based on just a few measurements conducted at evenly spaced intervals throughout the day (Goldstein and Kakihana 1977). For animals consuming alcohol, this approach can also be used to estimate blood alcohol concentrations (BACs) throughout the day based on a few (e.g., three) measurements taken over a period of 24 hours. Although this approach is not accurate enough for detailed chronobiological studies, it provides a relatively simple technique for studies with a more pharmacological focus (e.g., studies that assess the association of BACs with other physiological processes).

## The Influence of Lighting Conditions on Alcohol Consumption Patterns in Rodents

Researchers have long studied the circadian activity patterns of rodents. The first detailed description of the activity patterns of rats and their periodic nature was provided by Richter (1922), who recorded the animals’ spontaneous activity and its relationship to lighting conditions, temperature, and timing of food availability. Those studies found that rats are clearly nocturnal animals and that their nocturnal activity is particularly great if they are fed at the beginning of the dark period rather than at the beginning of the light period. Subsequent studies also determined that when given free access to food and water, rats and other rodents consume most of that food during the active night hours.

As scientists began to use rodents as animal models for human alcohol consumption, they also studied the animals’ diurnal alcohol consumption patterns. These studies frequently were motivated by the researchers’ need to identify environmental factors that influence the animals’ level of alcohol self-administration. In addition, researchers needed to establish whether the diurnal changes in BACs and the resulting physiological effects of alcohol in the animals adequately mirror those in human alcoholics.

The earliest investigations of diurnal consumption patterns determined that when the alcohol is provided in the form of solutions containing between 7.5 and 25 percent alcohol in water (either as the only source of fluids or in addition to drinking water), most of the alcohol consumption (i.e., approximately 65 to 75 percent) occurs during the active dark period (Freund 1970; Hatton and Vieth 1974). This consumption pattern is similar to that observed for normal drinking water, as further demonstrated by experiments comparing the drinking patterns of an alcohol-preferring line of rats (i.e., AA rats) and a non-alcohol-preferring line (i.e., ANA rats) (Eriksson 1972). In those experiments, all animals were kept in an environment with 12 hours of light (from 6:00 a.m. to 6:00 p.m.) and 12 hours of darkness and had unlimited access to food, water, and an alcohol solution. Under these conditions, animals from both strains showed three peaks of fluid consumption at around 6–7 p.m., 11 p.m., and 3–4 a.m. The only difference between the strains was that the AA animals primarily consumed the alcohol solution, whereas the ANA rats almost exclusively consumed water. This consumption pattern with three distinct peaks of fluid consumption during the dark phase also was confirmed in another strain of alcohol-consuming rats, called Sardinian alcohol-preferring rats (Agabio et al. 1996), and in C57BL mice (Millard and Dole 1983).

Thus, alcohol consumption in rats and other rodents normally follows a distinct circadian pattern that coincides with the animals’ general activity and consummatory behavior patterns. Experimental manipulation, however, allows researchers to induce alcohol consumption that is evenly distributed across the day. For example, when rats receive alcohol as part of a liquid diet that is the animals’ only source of food and fluids, the nocturnal pattern of alcohol consumption is greatly reduced (i.e., the animals consume large quantities of the diet both at night and during the day), possibly because the animals cannot obtain enough food during their normal feeding sessions to meet their nutritional needs (Freund 1970, Reidelberger et al. 1996). (Most liquid diets have a very sweet taste; consequently, rodents possibly consume those liquid diets throughout the 24-hour cycle because of their preference for drinking sweet solutions.) Accordingly, researchers conducting animal experiments must adjust the mode of alcohol delivery to the aim of their experiments (e.g., whether they want to achieve constant BACs throughout the day or whether they are interested in the consequences of fluctuating BACs).

### The Potential Role of the Pineal Gland in Circadian Patterns of Alcohol Consumption

Because numerous studies had confirmed the distinct nocturnal alcohol consumption pattern that normally prevails in rodents, scientists throughout the 1970s sought to identify the mechanisms underlying this pattern. These investigations focused on the pineal gland, which is located in the brain, and its primary hormone product, melatonin. This hormone is secreted into the bloodstream and distributed throughout the body, where it influences the actions of numerous other hormones as well as exerts other effects (e.g., appears to decrease skin pigmentation). The secretion of melatonin into the blood follows a marked diurnal pattern, with blood levels in humans approximately 10 times greater at night than during the day. These observations, together with findings that rats kept in constant darkness have larger pineal glands and exhibit greater activity of the melatoninforming enzyme (Geller 1971), suggest that the pineal gland and melatonin may play a role in determining diurnal drinking patterns.

To investigate this hypothesis, researchers have primarily used two approaches: (1) removing the pineal glands of laboratory animals (e.g., rats and hamsters) to eliminate the influence of the body’s own (i.e., endogenous) melatonin and/or (2) treating the animals with additional (i.e., exogenous) melatonin to determine the effects of increased melatonin levels. The results of these investigations, however, have been rather inconsistent and allow no firm conclusions about the role of the pineal gland and melatonin in determining diurnal drinking patterns of rodents. Moreover, most of the studies conducted in this area are rather old, and the issue has not been investigated further in recent years. The following paragraphs summarize the research results obtained to date.

Geller (1971) treated two rats that showed no preference for alcohol under a normal light-dark cycle with melatonin using daily injections for 2 weeks. In those animals, alcohol consumption increased and water consumption decreased over those 2 weeks. However, because this experiment involved only two rats, its value is limited (see Sinclair 1972).

Blum and colleagues (1973) compared the alcohol consumption patterns of rats whose pineal glands had been removed with those of control animals both in total darkness and under a normal light-dark cycle. When placed in total darkness, the control animals showed significantly increased alcohol consumption and reduced water consumption. The animals whose pineal glands had been removed, despite showing some increase in alcohol consumption, consistently drank less alcohol and significantly more water than did the control animals. These findings suggest that the pineal gland indeed modulates the darkness-induced alcohol preference in rats. Treatment with exogenous melatonin, however, did not alter the alcohol or water intake of the animals whose pineal glands had been removed, possibly because the melatonin levels achieved were not sufficient to affect alcohol-drinking behavior or because the animals were already consuming relatively high alcohol levels that could not be increased further.

Reiter and colleagues (1974) obtained similar results when conducting the corresponding experiments in hamsters, which generally show a greater preference for alcohol than do rats. Thus, removal of the pineal gland reduced the animals’ darkness-induced alcohol preference (as well as their alcohol preference during a normal light-dark cycle), although exogenous melatonin administration had no effect on the hamsters’ alcohol preference, again possibly because the animals’ alcohol preference already was relatively high or because melatonin is relatively ineffective as a hormone in hamsters.

Burke and Kramer (1974), however, found just the reverse effects of pineal gland removal and exogenous melatonin administration. In that study, removal of the pineal gland of rats did not significantly alter the animals’ alcohol preference, although animals without a pineal gland consumed somewhat less alcohol than did the control animals. Administration of exogenous melatonin to rats that had an intact pineal gland but exhibited a low alcohol preference, however, substantially increased the animals’ alcohol consumption.

Several factors may influence the outcome of studies evaluating the role of melatonin and the pineal gland and thus contribute to the discrepancies among the results. One of those factors may be the choice of animal models (Rudeen and Symmes 1981). Thus, studies conducted in rats found that exogenous melatonin enhanced alcohol consumption (Geller 1971; Smith et al. 1980), whereas in hamsters exogenous melatonin had no effect (Reiter et al. 1974) or even reduced alcohol consumption (Rudeen and Symmes 1981).

Another factor may be the timing of the melatonin administration. For example, Smith and colleagues (1980) noted that when they administered melatonin to rats during the dark phase of the daily cycle, the animals’ self-administration of alcohol increased significantly. When the melatonin was administered during the light phase, however, it had only a weak, statistically not significant effect on alcohol consumption. The investigators speculated that if melatonin is administered during the dark phase, when the animals’ internal melatonin levels are already high, the additional melatonin may boost overall hormone levels above the limits needed to achieve an effect. Conversely, if the melatonin is administered during the light phase, overall hormone levels may still be too low to promote alcohol consumption. Accordingly, researchers must consider such issues when designing their experiments.

Burke and Kramer (1974) speculated on potential mechanisms through which the exogenous melatonin could influence alcohol consumption. First, melatonin itself or one of its breakdown products may interact with alcohol or its breakdown product (i.e., acetaldehyde) to form chemicals that may stimulate alcohol preference. Second, exogenous melatonin might alter the normal levels of the brain chemical (i.e., neurotransmitter) serotonin, which may play a role in tolerance, withdrawal, and intoxication. Serotonin is a precursor for melatonin—that is, through several metabolic steps, serotonin is converted to melatonin in the pineal gland. Administration of exogenous melatonin may reduce the body’s own rate of melatonin formation, thereby resulting in elevated serotonin levels, which in turn might influence alcohol preference.

This relationship between melatonin levels, serotonin levels, and alcohol preference was further investigated by Geller and Hartman (1977), who treated hamsters with a serotonin precursor called 5-hydroxytryptophan (5–HTP). When the animals received 5–HTP injections for several days, alcohol consumption declined substantially in some of the animals, suggesting that changes in serotonin levels and/or the resultant changes in melatonin levels may influence alcohol consumption patterns in hamsters.

## Factors Influencing the Effects of Lighting Conditions on Alcohol Consumption

More recently, researchers have focused on the analysis of various factors that might influence diurnal drinking patterns of laboratory animals. Such factors include strain differences; differences in the type of alcohol-containing diet or its administration schedule; disruption of the normal daily light-dark cycle; concurrent administration of other drugs; the body’s own signaling systems, such as hormones; and the properties (e.g., wavelength) of the light used.

### Strain Differences

As mentioned earlier in this article, substantial differences in alcohol consumption patterns exist among various types of rodents and even among different strains of the same type of rodent. For example, hamsters generally have a higher preference for alcohol than do many commonly used strains of rats (Reiter et al. 1974; Rudeen and Symmes 1981). Similarly, some rat strains have a significantly higher preference for alcohol than do other strains (Eriksson 1972; Aalto 1986).

Strain differences also may exist in the specific diurnal consumption patterns. For example, although initial analyses of the alcohol-preferring AA rats and the nonalcohol-preferring ANA rats had indicated that both strains had similar consumption patterns of alcohol and water (Eriksson 1972), detailed analyses using more sophisticated measuring techniques subsequently demonstrated that some differences do exist (Aalto 1986). Thus, the AA rats showed three peaks of alcohol consumption during the dark period that coincided with smaller peaks in water consumption. Conversely, the ANA rats exhibited two major peaks in water consumption during the dark phase and rather evenly distributed, low-level alcohol consumption throughout the dark phase.

Similarly, a comparison of two rat strains called Fisher rats and spontaneous hypertensive (SP) rats found that the SP rats demonstrated clear diurnal consumption patterns for both alcohol and water, regardless of whether the animals were kept on a normal light-dark cycle or in complete darkness (Pasley et al. 1987). Conversely, the Fisher rats exhibited a circadian consumption pattern only for water when they were kept under a light-dark cycle. The animals exhibited no such pattern, however, for water when they were kept in complete darkness or for alcohol under any lighting condition (i.e., their alcohol and water consumption under these conditions remained constant throughout the day).

Although such differences in alcohol consumption patterns might seem trivial, for some experiments researchers should be familiar with the consumption patterns of the specific animal strains they are using, because those patterns also determine the animals’ BACs throughout the day. For example, if investigators wish to determine the animals’ peak BACs (e.g., to study whether those BACs are high enough to cause intoxication or other behavioral effects), they must know when to take the blood samples. Accordingly, they also must know when the greatest alcohol consumption occurs, because BACs are likely to be highest shortly afterwards.

**Figure 1:**
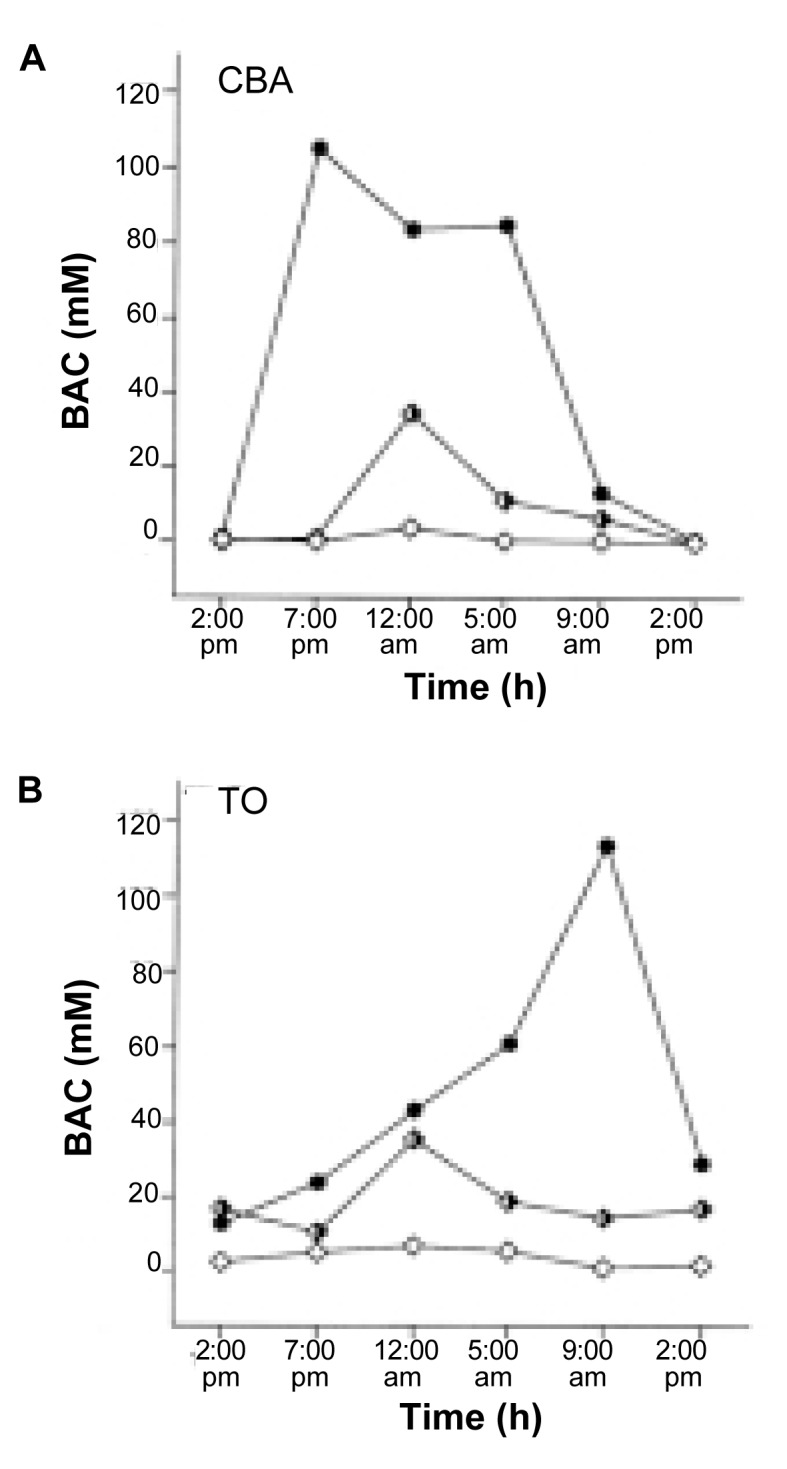
Alcohol consumption patterns of two mouse strains (CBA and TO) that received alcohol either in a liquid diet containing 7-percent alcohol that constituted the animals’ sole source of food or as an alcohol solution in addition to regular laboratory food. When the alcohol was provided in the form of a liquid diet (circles), the alcohol consumption patterns (and thus the blood alcohol concentrations [BACs]) of the two mouse strains differed considerably. In the CBA mice (A), BACs peaked around 7 p.m. and remained high throughout the night. Conversely, the BACs in TO mice (B) showed a sharp peak around 9 a.m. When the alcohol was provided in the form of a solution containing 10 percent (empty squares) or 20 percent (half-empty squares) alcohol, however, alcohol consumption and BACs in both strains were considerably reduced and distributed more evenly throughout the day. Furthermore, no substantial differences existed in the alcohol consumption patterns of the two strains under these conditions. (Error bars are not shown.) mM = millimolar. SOURCE: Jelic et al. 1998.

For example, Jelic and colleagues (1998) compared two mouse strains called CBA and TO that received an alcohol-containing liquid diet^2^ as their only source of food and were kept under the same environmental conditions. The investigators found that the CBA mice exhibited their peak BACs around 7 p.m. and that BACs remained relatively high until 5 a.m. (see figure 1). Conversely, for the TO mice the peak BACs were observed at 9 a.m. and dropped rapidly afterwards. Thus, to determine peak BACs, researchers would need to take blood samples from the animals at different times of the day.

### Differences in the Type and Administration Schedule of the Alcohol-Containing Diet

Other factors that influence the alcohol consumption of laboratory animals, including the circadian consumption patterns, are related to the way in which the alcohol is provided. Most rats and mice are neophobic—that is, they fear changes in their environment (including diet)—and appear to find alcohol aversive at first. As a result, most rodents initially are reluctant to consume alcohol when offered a choice between alcohol-containing solutions and water. To motivate the animals to consume alcohol, researchers have used various approaches. For example, they have offered alcohol-containing solutions as the only fluid, allowed the animals access to liquids (i.e., alcohol and/or water) for only a short time each day, maintained the animals on an alcohol-containing liquid diet as their sole food, or sweetened and salted the alcohol solution (Kulkosky 1979).

The approach that researchers choose to motivate study animals to consume alcohol (and the alcohol concentration in the respective fluids) can substantially alter overall alcohol intake and diurnal consumption patterns. For example, Jelic and colleagues (1998) compared the alcohol consumption patterns (and resulting BACs) of CBA and TO mice that were maintained either on a diet consisting of regular laboratory food plus alcohol solution as the sole fluid or on an alcohol-containing liquid diet. The investigators found that animals of both strains consumed less alcohol (and therefore experienced lower BACs) when they received the alcohol in their drinking water. Moreover, when the alcohol was provided in the drinking water, fluid consumption was more evenly distributed throughout the day and/or peaks were lower and occurred at different times than when the alcohol was part of a liquid diet (see figure 1).

**Figure 2:**
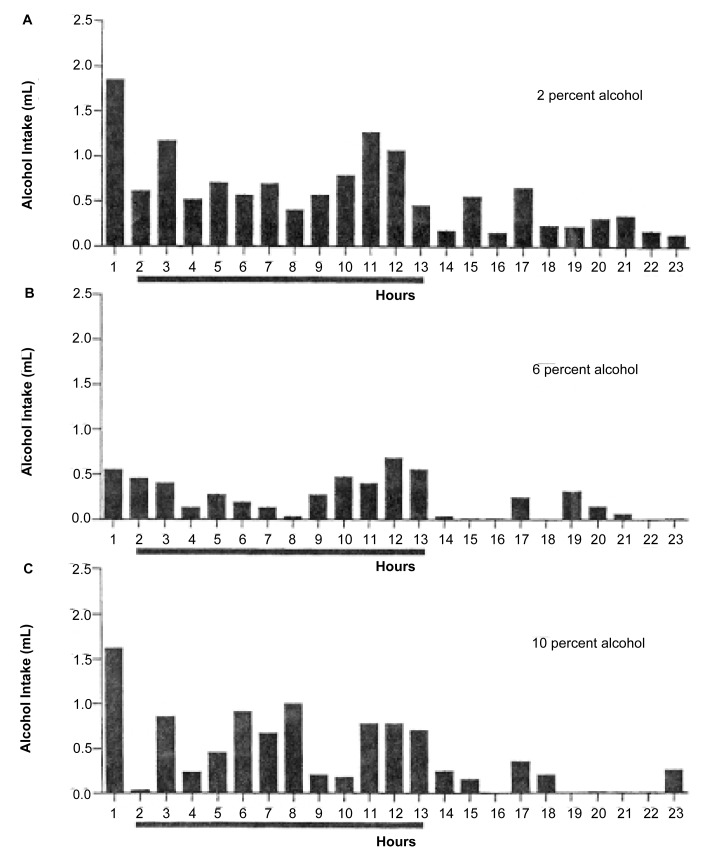
Alcohol consumption patterns of rats receiving drinking water containing (A) 2 percent, (B) 6 percent, or (C) 10 percent alcohol. The animals had access to the alcohol solution for 23 hours per day. Both overall fluid consumption and circadian consumption patterns differed depending on the alcohol concentration in the drinking water. The horizontal dark line indicates the dark period in the animals’ light-dark cycle. (Error bars are not shown.) mL= milliliter. SOURCE: Boyle et al. 1997.

The concentration of the alcohol solution offered also can influence consumption patterns. In one study, rats were offered alcohol in increasing concentrations ranging from 2 to 10 percent in their drinking water (Boyle et al. 1997). The animals had access to the drinking solution for 23 hours per day, after which it was withheld for 1 hour. Slight variations in the circadian consumption patterns existed with different alcohol concentrations. Thus, with most concentrations, the animals consumed a relatively large amount of alcohol in the hour after they regained access to the alcohol solution. With the 6-percent alcohol solution, however, the animals drank a substantially smaller amount during that first hour than with the other alcohol concentrations. Furthermore, whereas with most alcohol solutions the majority of the consumption occurred during the dark phase, a relatively high level of consumption occurred during the light phase with the 2-percent solution. Finally, the number and intensity of consumption peaks varied for different alcohol solutions (see figure 2). These observations indicate that the concentration of the alcohol solution offered can influence circadian consumption patterns in rodents.

The schedule with which alcohol is available to the animals also influences their drinking behavior. Thus, in animals that have continuous access to alcohol, consumption occurs in numerous discrete drinking bouts that are distributed mainly throughout the dark phase. When access to alcohol is limited to a short period of time during the light phase each day, however, the animals will consume substantial amounts even at that “unnatural” time (Gill et al. 1986). In fact, with such a limited access procedure, alcohol intake can result in detectable alcohol levels in the blood and brain as well as in significant behavioral changes.

### Disruption of the Normal Circadian Rhythm

As indicated by the studies described in the previous sections, food and fluid intake (including alcohol consumption) in rodents (and probably also in humans) is strongly influenced by the normal circadian cycle. Many people, however, frequently experience shifts in their normal daily cycles (e.g., shift workers and people traveling across time zones). Researchers have been interested in determining how such “phase shifts” influence drinking behavior and have designed some animal models accordingly. For example, Choi and colleagues (1990) maintained rats on a limited access schedule that allowed them access to a sweetened alcohol solution and to water for only 2 hours per day. This access period began either shortly after the start of the dark period, representing a normal circadian rhythm, or shortly after the start of the light period, representing a disrupted circadian rhythm. The investigators found that after an adjustment period of approximately 3 weeks, during which animals with a normal circadian rhythm consumed more alcohol, the situation was reversed and the animals with the disrupted circadian rhythm reliably showed greater consumption. Furthermore, the animals with the normal circadian rhythm seemed to thrive better (as indicated by their weight gain throughout the study) than did the animals with the disrupted circadian rhythm. These observations suggest that disruption of the circadian rhythm may result in increased alcohol intake and may interfere with overall well-being.

Other investigators studied the effects of a disrupted circadian cycle by offering alcohol solutions to rats kept under constant light or constant darkness (Goodwin et al. 1999). This analysis found that both during an acquisition phase, in which the animals became accustomed to alcohol, and during a subsequent maintenance phase, in which the animals had constant access to alcohol, exposure to constant light suppressed the animals’ alcohol consumption compared with animals kept under normal lighting conditions. In contrast, exposure to complete darkness during both the acquisition and the maintenance phase did not substantially affect the rats’ alcohol consumption compared with animals kept under normal lighting conditions. Furthermore, the different lighting conditions did not influence the animals’ water consumption. These findings suggest that certain lighting conditions can specifically affect alcohol intake.

The influence of a disrupted circadian rhythm with altered lighting conditions was further investigated by Gauvin and colleagues (1997), who altered the lighting conditions of rats in a manner that reflected the “jet lag” experienced by travelers crossing several time zones and the repeated changes in schedule experienced by shift workers. In those experiments, animals exposed to the jet lag conditions showed temporary increases in alcohol consumption. Moreover, animals kept under shift work conditions demonstrated significantly increased alcohol intake over a 2-month testing period. These findings further support the notion that a disruption of the normal circadian rhythm can result in increased alcohol consumption.

### Concurrent Administration of Other Drugs

Some alcohol users also take other drugs, and scientists have mimicked this condition in animal experiments. For example, in the study by Choi and colleagues (1990) mentioned in the previous section, the investigators continuously treated some of the animals with morphine and studied the effects of this treatment on the alcohol consumption of rats with a normal or a disrupted circadian cycle. These analyses found that alcohol consumption and preference for alcohol over water was greatest in those animals that both were exposed to morphine and experienced a disrupted circadian cycle. Even among the animals with a normal circadian cycle, treatment with morphine increased alcohol consumption and alcohol preference. This finding contrasts with other observations that acute morphine injection can strongly suppress voluntary alcohol consumption by rats and hamsters (for a review, see Sinclair 1980).

Choi and colleagues (1990) speculated that among other mechanisms, brain chemicals called endogenous opioids may play a role in the relationship between morphine administration, disruption of the circadian cycle, and alcohol consumption. The activity of these substances changes across the daily cycle, and disruptions in the circadian rhythm may perturb the functions of the endogenous opioids. Morphine is chemically related to the endogenous opioids and therefore also affects brain functions controlled by the opioids. Consequently, morphine administration may exacerbate the effects of a disrupted circadian cycle on endogenous opioid function.

### Signaling Systems in the Body

The body’s endogenous signaling systems, such as certain hormones or neuropeptides—that is, small protein molecules that relay information to nerve cells—also control alcohol intake. One such hormone and neuropeptide called cholecystokinin (CCK) is released in the gastrointestinal tract and brain and appears to serve as a signal of satiety. For example, researchers have demonstrated that exogenous CCK suppresses feeding and alcohol intake in rodents (Kulkosky et al. 1991).

The magnitude of this effect appears to be dependent on whether CCK is administered and alcohol is available during the dark phase or the light phase of the daily cycle. To demonstrate this interaction between CCK and the lighting cycle in reducing alcohol intake, Kulkosky and colleagues (1991) used a limited-access procedure in which rats had access to alcohol for only 40 minutes per day either during the dark phase or during the light phase. Immediately before each access period, the animals were injected with CCK. The investigators found that CCK limited alcohol intake by a greater proportion compared with the control animals when CCK administration and alcohol access occurred during the dark phase than when they occurred during the light phase. These observations are consistent with those of other researchers who noted that another gastrointestinal neuropeptide called bombesin also appeared to inhibit feeding more potently at night (Kulkosky et al. 1991). However, for both bombesin and CCK, the details of the experimental design appear to influence to a certain extent whether they are more effective during the dark or during the light phase (Kulkosky et al. 1991).

### Properties of Light

One line of research into the factors that influence the relationship between alcohol consumption and lighting conditions has addressed the issue of whether the wavelength of light used plays any role in this relationship. These studies were triggered by observations that rats whose cages were placed by a window had a greater preference for alcohol than did rats whose cages were placed at other locations in the laboratory (Wilson et al. 1976). Because initial studies detected no effect of light intensity (which would be greater by the window) on alcohol consumption, Wilson and colleagues (1976) investigated the effect of ultraviolet (UV) light (the levels of which also should be higher near the window) on alcohol preference.

In that experiment, rats were kept under diffuse white light for 12 hours per day for 10 days before one-half of the animals were placed under UV light^3^ for 12 hours per day for 30 days. Within 3 days of placing the animals under UV light, their alcohol consumption increased markedly, accompanied by a substantial decrease in water consumption. This increase in alcohol consumption was not permanent, however. When the UV light was removed, the animals’ alcohol consumption slowly decreased to its normal levels over a period of 30 to 40 days. Conversely, the alcohol and water consumption of the control animals remained stable throughout the experimental period. The mechanism through which UV light exerts its effect on alcohol preference still remains unclear, although it is known that UV light potentiates the toxicity of other ingested chemicals.

## Conclusions

Researchers have studied the relationship between the light-dark cycle and alcohol consumption for approximately 30 years and have uncovered solid evidence that at least in rodents, the lighting conditions influence normal drinking behavior. Furthermore, scientists have identified several factors that modulate this relationship. Despite these extensive investigations, however, the field is still evolving and many questions remain. For example, the role of the pineal gland and its hormone melatonin in governing alcohol consumption has not been determined conclusively. Other topics, such as the properties (e.g., wavelength) of the light that may play a role in regulating alcohol consumption, have not yet received the attention they deserve.

## Footnotes

1 The term “circadian rhythm” refers to any pattern that is based on an (approximately) 24-hour cycle. Conversely, the term “diurnal rhythm” refers to patterns of activity or behavior that follow day-night cycles, such as the breakfast-lunch-dinner schedule.

2 The diet contained 3.5-percent alcohol (comparable to the alcohol content in beer) for 2 days, then 7-percent alcohol for 5 days.

3 The wavelength of the ultraviolet light (i.e., 350 nm) was such that it was not harmful to the animals in any way.
